# Marine-Inspired Enzymatic Mineralization of Dairy-Derived Whey Protein Isolate (WPI) Hydrogels for Bone Tissue Regeneration

**DOI:** 10.3390/md18060294

**Published:** 2020-06-02

**Authors:** Karl Norris, Magdalena Kocot, Anna M. Tryba, Feng Chai, Abdullah Talari, Lorna Ashton, Bogdan V. Parakhonskiy, Sangram K. Samal, Nicholas Blanchemain, Elżbieta Pamuła, Timothy E. L. Douglas

**Affiliations:** 1 Engineering Department, Lancaster University, Lancaster LA1 4YW, UK; a.talari@lancaster.ac.uk (A.T.); t.douglas@lancaster.ac.uk (T.E.L.D.); 2 Department of Biomaterials and Composites, Faculty of Materials Science and Ceramics, AGH University of Science and Technology, 30-962 Kraków, Poland; kocotmagda@gmail.com (M.K.); amtryba@agh.edu.pl (A.M.T.); epamula@agh.edu.pl (E.P.); 3 INSERM U1008-Controlled Drug Delivery Systems and Biomaterials, Université de Lille, 59006 Lille, France; fchai@univ-lille2.fr (F.C.); nicolas.blanchemain@univ-lille.fr (N.B.); 4 Chemistry Department, Lancaster University, Lancaster LA1 4YW, UK; l.ashton@lancaster.ac.uk; 5 Department of Biotechnology, Ghent University, B-9000 Gent, Belgium; Bogdan.parakhonskiy@ugent.be; 6 Nanotechnology Department, Saratov State University, Saratov 410012, Russia; 7 Laboratory of Biomaterials and Regenerative Medicine for Advanced Therapies, Indian Council of Medical Research-Regional Medical Research Center, Bhubaneswar, Odisha 751023, India; sksamalrec@gmail.com; 8 Materials Science Institute (MSI), Lancaster University, Lancaster LA1 4YW, UK

**Keywords:** hydrogel, composite, mineralization, enzyme, bioinspired, whey protein isolate

## Abstract

Whey protein isolate (WPI) is a by-product from the production of cheese and Greek yoghurt comprising β-lactoglobulin (β-lg) (75%). Hydrogels can be produced from WPI solutions through heating; hydrogels can be sterilized by autoclaving. WPI hydrogels have shown cytocompatibility and ability to enhance proliferation and osteogenic differentiation of bone-forming cells. Hence, they have promise in the area of bone tissue regeneration. In contrast to commonly used ceramic minerals for bone regeneration, a major advantage of hydrogels is the ease of their modification by incorporating biologically active substances such as enzymes. Calcium carbonate (CaCO_3_) is the main inorganic component of the exoskeletons of marine invertebrates. Two polymorphs of CaCO_3_, calcite and aragonite, have shown the ability to promote bone regeneration. Other authors have reported that the addition of magnesium to inorganic phases has a beneficial effect on bone-forming cell growth. In this study, we employed a biomimetic, marine-inspired approach to mineralize WPI hydrogels with an inorganic phase consisting of CaCO_3_ (mainly calcite) and CaCO_3_ enriched with magnesium using the calcifying enzyme urease. The novelty of this study lies in both the enzymatic mineralization of WPI hydrogels and enrichment of the mineral with magnesium. Calcium was incorporated into the mineral formed to a greater extent than magnesium. Increasing the concentration of magnesium in the mineralization medium led to a reduction in the amount and crystallinity of the mineral formed. Biological studies revealed that mineralized and unmineralized hydrogels were not cytotoxic and promoted cell viability to comparable extents (approximately 74% of standard tissue culture polystyrene). The presence of magnesium in the mineral formed had no adverse effect on cell viability. In short, WPI hydrogels, both unmineralized and mineralized with CaCO_3_ and magnesium-enriched CaCO_3_, show potential as biomaterials for bone regeneration.

## 1. Introduction

Natural by-products from industrial processes have tremendous biomimetic properties and are inexpensive as they are infrequently utilized. Whey protein isolate (WPI) is a by-product from the production of cheese and Greek yoghurt comprising β-lactoglobulin (β-lg) (50%) and α-lactalbumin (α-la) (20%) [1]. WPI in solution has been shown to enhance osteogenic differentiation of bone-forming cells and promote cellular proliferation [1]. It was recently discovered that hydrogels can be produced from WPI solutions, whereby gelation is achieved through heating and sterilization is achieved by autoclaving [2,3,4].

With regards to mechanical properties, hydrogels are relatively weak due to the high water content. However, the incorporation of a mineral phase may improve the mechanical properties and therefore, promote cellular adhesion, proliferation and osteogenic differentiation [5]. In comparison to ceramic minerals, a major advantage of hydrogels is that they can be modified with ease by incorporating biologically active substances such as enzymes. Similarly, it is possible to modify and enhance properties of hydrogels by enriching the solution with a mineral phase either before or after gelation [6].

Marine invertebrates use the mineral calcium carbonate (CaCO_3_) in their exoskeletons. CaCO_3_ occurs naturally as three crystalline polymorphs known as calcite, aragonite and vaterite. Calcite occurs in bivalves and certain sponges, while aragonite occurs in coral and nacre, or “mother of pearl”. All three polymorphs of CaCO_3_ may exhibit beneficial biological properties. For example, calcite is a bioactive substance that can form direct connections with bone tissue in vivo and could potentially strengthen fractured bone [7]. Aragonite has been employed as a biomaterial for bone regeneration and outperformed calcium phosphates [8], and when added in particle form to WPI hydrogels, has improved mechanical properties and proliferation of osteoblast-like cells [4]. Vaterite coatings have been shown to stimulate the formation of apatite upon incubation in simulated body fluid (SBF) [9]. Hence, one may hypothesize that enzymatic mineralization of WPI hydrogels with polymorphs of CaCO_3_ will improve their biological performance in vitro and in vivo.

Several approaches to mineralize hydrogels have been tried (Gkioni et al—Reference 5). The most popular approach is the addition of pre-formed ceramic particles (e.g., hydroxyapatite, bioactive glasses) during hydrogel formation. Enzymatic mineralization has certain advantages over addition of pre-formed particles. Firstly, particles are prone to aggregation, resulting in inhomogeneous distribution, while enzymatic mineralization can potentially lead to a more homogeneous distribution of the mineral and better integration of the mineral with the hydrogel network. Secondly, the amount of pre-formed ceramic particles which can be incorporated is limited to approximately 30–50% [5], as the presence of too many particles may impede formation of the hydrogel. Using enzymatic mineralization, larger mineral contents can be achieved.

In previous work, hydrogels have been mineralized enzymatically with calcium phosphate (CaP) using alkaline phosphatase (ALP), the enzyme responsible for mineralization of bone tissue. One advantage of using urease instead of ALP is the superior thermal stability of urease at higher temperatures such as 70 °C, at which WPI hydrogels are formed.

The reaction steps of mineral precipitation using urease have been described previously [10]. Briefly, urea diffuses into the hydrogel, where it is converted under the action of urease into ammonia and bicarbonate ions. After dissociation of bicarbonate ions to hydrogen and carbonate ions, the carbonate ions react with calcium, which has also diffused into the hydrogel, to form CaCO_3_. The hydrogen ions formed by the dissociation of the bicarbonate ions are neutralized by the ammonia, resulting in a sufficiently high pH to allow CaCO_3_ precipitation.

Another beneficial enriching agent is magnesium, which can be found in the calcareous exoskeletons of certain marine organisms and may provide benefits to mammals [11]. Previous work has shown that the presence of magnesium in CaP has promoted adhesion and proliferation of osteoblastic cell lines [12]. During enzymatic mineralization of hydrogels with CaCO_3_, magnesium ions can be added to the mineralization medium which may lead to an increase in cell number [13,14]. In previous work, Gellan Gum (GG) hydrogels have been mineralized with CaCO_3_ and/or calcium magnesium carbonate by incorporation of the enzyme urease into the hydrogel network followed by incubation in a solution containing the enzyme substrate, urea, and calcium and magnesium ions [14,15].

In the following study, the enzymatic mineralization approach was extended to WPI hydrogels. WPI hydrogels were mineralized with CaCO_3_ or magnesium-enriched CaCO_3_ via enzymatic mineralization with urease and incubation in solutions containing calcium and magnesium ions. The novelty of this study lies in both the enzymatic mineralization of WPI hydrogels and enrichment of the mineral with magnesium. It was thus demonstrated that urease retained activity after incorporation into WPI hydrogels, which involved gelation at 70 °C. Three different Ca:Mg concentration ratios were compared. The resulting hydrogels were subjected to physicochemical, morphological and biological characterization to examine the influence of Ca:Mg ratio.

## 2. Results

### 2.1. Influence of Mineralization Medium on Extent and Elemental Composition of Mineral Formed

**Figure 1:**
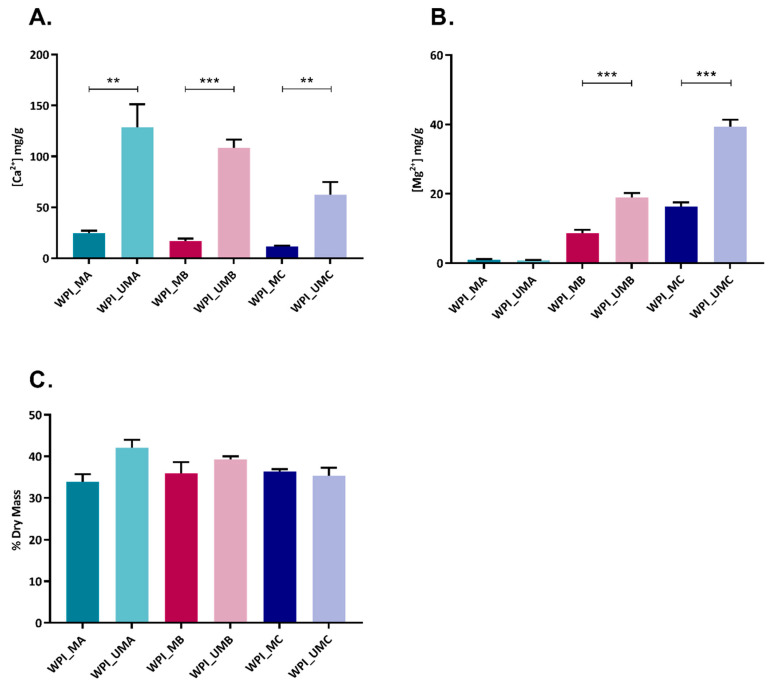
Mass of elemental calcium (**A**) and magnesium (**B**) per unit mass of hydrogel and dry mass percentage (**C**) of WPI hydrogels without urease (WPI) and with urease (WPI_U) incubated in different media: MA, MB, MC, *n* = 3, ** *p* < 0.01, *** *p* < 0.001.

We aimed to enhance the properties of WPI hydrogels by mineralization with CaCO_3_ or magnesium-enriched CaCO_3_. The effects of Ca^2+^ and Mg^2+^ content of the mineralization media on extent of mineral formed were investigated. As expected, a dose dependent effect was observed depending in which medium the urease WPI hydrogel was incubated (Table 1, Figure 1A,B). Notably, WPI hydrogels containing urease were able to retain higher amounts of Ca^2+^ and Mg^2+^ than control hydrogels, suggesting a greater extent of mineralization. When WPI hydrogels containing urease were incubated with calcium and magnesium in equimolar concentrations (WPI_U_MC), calcium content was five-fold that of magnesium, indicating that calcium was preferentially incorporated into the hydrated polymer. The increase in dry mass as a result of enzymatic mineralization was highest for hydrogels incubated in calcium only (WPI_U_MA) (Figure 1C).

### 2.2. Physicochemical Characterization of Mineral Formed by FTIR, Raman, XRD and SEM

**Figure 2:**
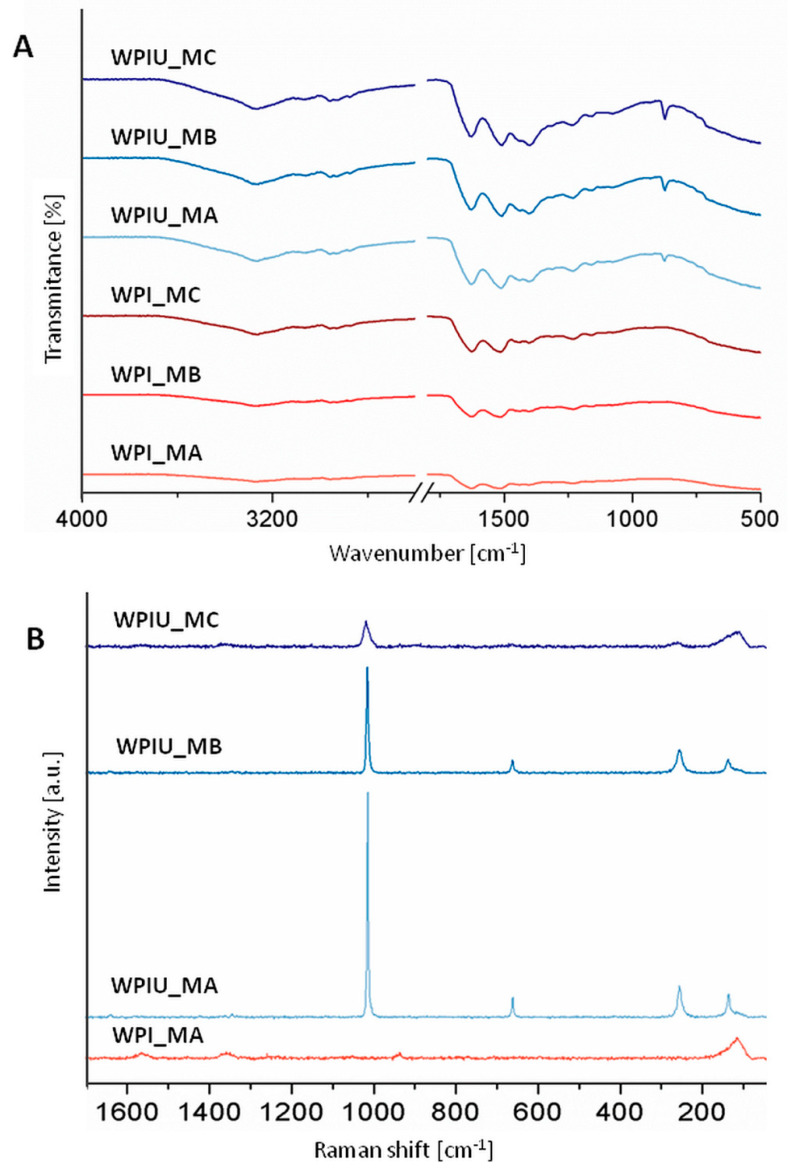
FTIR (**A**) and Raman spectra (**B**) of WPI hydrogels without and with urease WPI_U incubated in different media: MA, MB, MC.

FTIR spectra (Figure 2A) of urease-free hydrogels indicated characteristic bands for WPI at 1650, 1570 and 1350–1200 cm^−1^ [16]. In contrast, hydrogels containing urease showed bands characteristic of calcium carbonate. There was a broad band at 1400 cm^−1^, which is indicative of calcite and corresponds to *v_3_* antisymmetric stretching of the carbonate group [17]. Moreover, a band at approximately 1500 cm^−1^ was detected, which might indicate the presence of vaterite. This band was more intense in WPI_U_MA than in samples incubated in MB and MC. A band at approximately 1080 cm^−1^ was also observed in all hydrogels with urease and might indicate that vaterite was formed within these hydrogels [18]. In all hydrogels with urease, bands at 870 and 715 cm^−1^ correspond to *v_2_* out-of-plane bending and *v_4_* in-plane bending, respectively. These bands are characteristic for calcite [17]. These results show strong evidence that, in WPI hydrogels containing urease, the minerals calcite and vaterite were formed.

Raman spectra of control WPI_MA hydrogels (Figure 2B) showed bands at 1658 and 1453 cm^−1^, which are associated with amide I and CH_2_ bending, respectively [19]. There were also bands at approximately 1000 cm^−1^ relating to phenylalanine [20,21]. Bands typical for CaCO_3_ were not observed. In contrast, WPI_U_MA and WPI_U_MB showed intense bands at 1085 cm^−1^, which are typical for CaCO_3_ and correspond to *v_1_* symmetric stretching [22]. Raman bands were also observed at 711 cm^−1^ relating to *v_4_* in-plane stretching and both hydrogels showed bands at 281 and 154 cm^−1^ consistent with lattice vibration modes [22]. Sharp peaks in WPI_U_MA and WPI_U_MB were indicative of crystalline structure. In WPI_U_MC, Raman bands at 1085 cm^−1^ were broader and less intense, suggesting increased amorphicity, and thus, incorporation of magnesium in the calcite lattice. It was found that increased magnesium concentration reduced the crystallinity of CaCO_3_. This observation was confirmed by two other bands typical for calcite (at 281 and 154 cm^−1^), which were also broader in WPI_U_MC than in WPI_U_MA and WPI_U_MB [17,23,24]. The decrease in intensity and band sharpness in the order WPIU_MA > WPIU_MB > WPIU_MC suggests a decrease in crystallinity in the same order.

**Figure 3:**
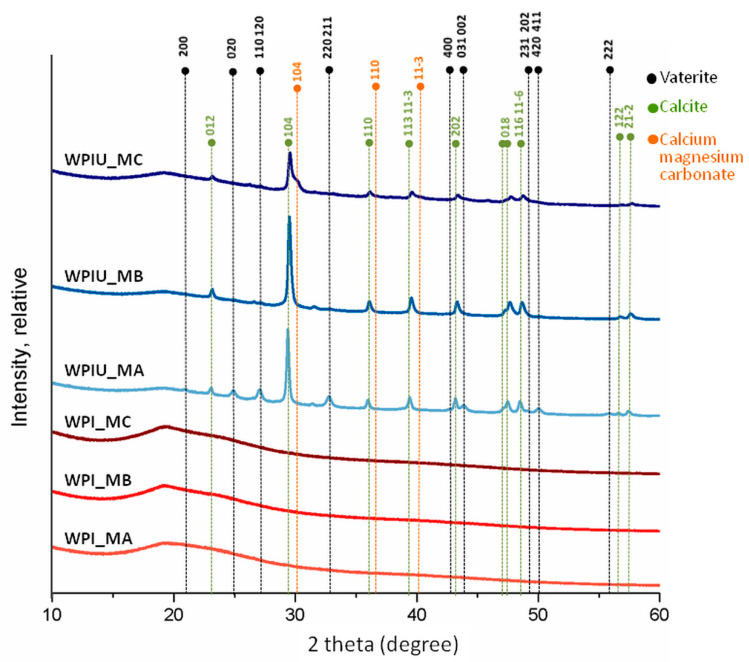
XRD diffractograms of WPI hydrogels incubated in different media MA, MB and MC.

XRD diffractograms of control samples (urease-free hydrogels) displayed a peak at a 2 theta value of approximately 19.5, which is a characteristic peak for WPI (Figure 3) [20]. However, in WPI hydrogels with urease (WPI_U), this peak was less intense, which might suggest that the proportion of mineral present in this sample was higher than the proportion of WPI. Minerals formed in all WPI hydrogels containing urease, displaying peaks at 23.2, 29.5, 36.1, 39.5, 43.3, 47.7, 48.6 and 57.5, which are characteristic for calcite [13,25]. In addition, WPI_U_MA hydrogels displayed peaks characteristic for vaterite at 24.9, 27.0 and 32.8 [26]. A peak near 30 degrees in WPI_U_MC hydrogels may indicate the presence of calcium magnesium carbonate. XRD may be also used to identify the degree of crystallinity of material. Crystalline materials show sharp peaks whereas amorphous produce a broad background pattern. Therefore, it could be concluded that WPI hydrogels with urease incubated in media MA and MB (WPIU_MA and WPIU_MB) are crystalline, as indicated by sharp peaks [13,25]. The lower intensity and sharpness of peaks of hydrogels incubated in medium MC (WPI_U_MC) suggest lower crystallinity.

**Figure 4:**
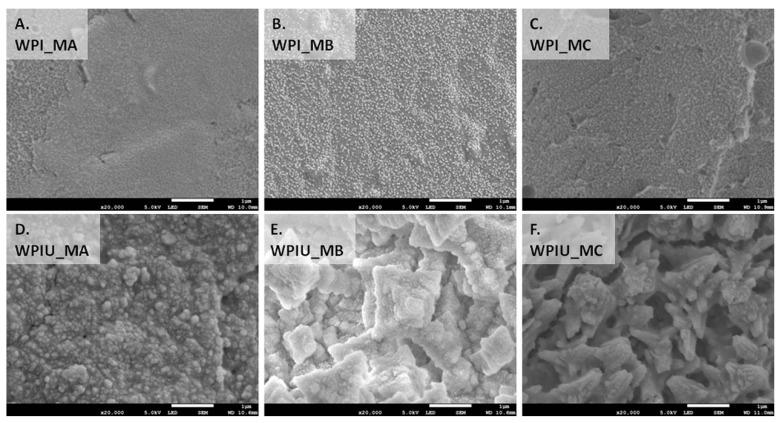
SEM images of WPI and WPI_U hydrogels incubated in media MA (**A**,**D**), MB (**B**,**E**) and MC (**C**,**F**). Scale bar is representative of 1 μm (**A**,**B**,**C**) and 10 μm (**D**,**E**,**F**).

The morphology of the different WPI hydrogels was studied by SEM (Figure 4). As expected, WPI hydrogels without urease displayed surfaces devoid of mineral deposits (Figure 4A–C). The surface of hydrogels incubated in medium MA was smooth, whereas hydrogels incubated in media MB and MC had some deposits on the surface, which might have been polymer residues. In contrast, WPI hydrogels containing urease displayed microstructures rich in mineral deposits (Figure 4D–L). In mineralized hydrogels incubated in medium MA (WPI_U_MA), some porous cube-like deposits typical for calcite and spherical deposits typical for vaterite were observed [27,28]. Moreover, some undefined elongated, rod-like deposits were also detected on the surface of hydrogels incubated in medium MA. Cuboid deposits typical for calcite were also observed in WPI hydrogels incubated in media containing magnesium (MB and MC). However, there were also some undefined deposits in samples incubated in medium with the highest concentration of magnesium (MC), which were not described in previous studies [14,27]. This finding suggests a lower degree of crystallinity of the mineral formed in such samples (WPI_U_MC). In mineralized hydrogels incubated in medium MB (WPI_U_MB), besides rhombohedral crystals of calcite, there were also rod-like crystals covered with spherical deposits. Plate-like deposits, which were reported in a previous work on GG hydrogels and are characteristic of hydromagnesite, were not observed in WPI hydrogels [14].

### 2.3. Cytotoxicity Studies and Release of Ca and Mg into Cell Culture Medium

**Figure 5:**
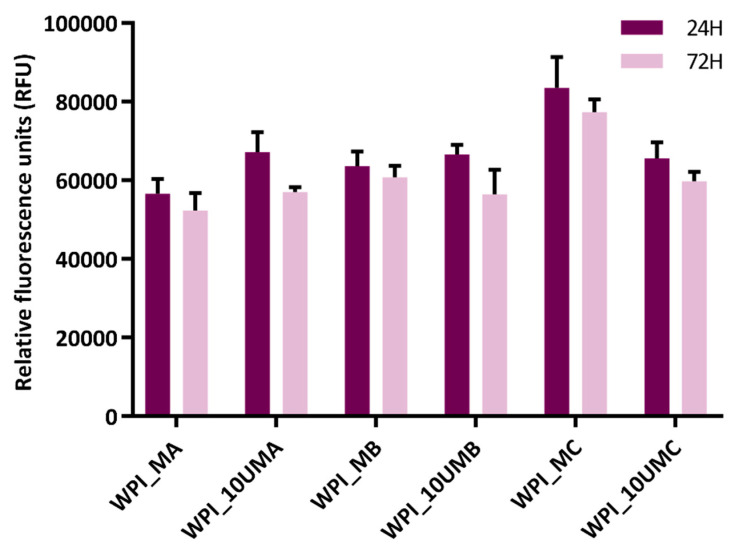
Cell viability of mouse osteoblast (MC3T3-E1 cells) seeded onto hydrogels over 24 h and 72 h. Background fluorescence of AlamarBlue and cell culture media was subtracted from each experimental hydrogel. *n* = 3, error bars are representative of SD.

Cell viability experiments utilizing the AlamarBlue assay (Figure 5) indicated that none of the hydrogels were immediately cytotoxic. Cells were viable following 24 h incubation on the WPI hydrogels. Interestingly, cell viability increased on WPI hydrogels which lacked urease where the calcium and magnesium concentrations decreased and increased, respectively (WPI_MA vs. WPI_MB vs. WPI_MC). In contrast, viability remained similar for all WPI hydrogels mineralized with urease, independent of the mineralization medium used. For the most part, cell viability remained comparable after 72 h, indicating that the cells were able to adhere and remain viable.

**Figure 6:**
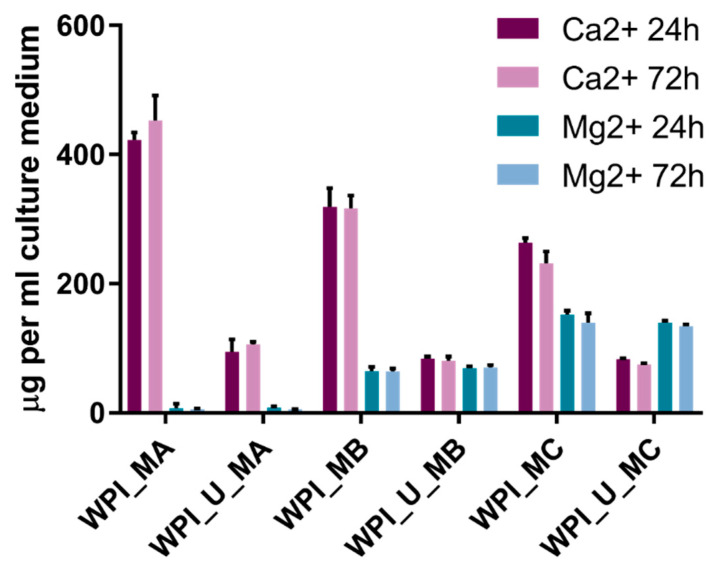
Concentrations of elemental Ca and Mg in hydrogel extracts after 24 h and 72 h. *n* = 3, error bars show standard deviation.

The results of measurements of elemental Ca and Mg in extraction medium (Figure 6) revealed that considerably more Ca and Mg were released from hydrogels which did not contain urease, i.e., which were unmineralized. One explanation may be the presence of residual mineralization medium containing Ca and Mg in the hydrogels, allowing these ions to diffuse out more easily from unmineralized samples. In mineralized samples, taking into account the lower amount of Mg present compared to Ca (see methods and materials; Table 1), Mg was preferentially released. It was noticeable that the concentrations present in the extraction medium were similar after 24 h and 72 h. This suggests that practically all Ca and Mg release takes place within the first 24 h.

## 3. Discussion

Enzymatic mineralization of WPI hydrogels was demonstrated directly by FTIR and Raman spectra (Figure 2), XRD diffractograms (Figure 3), SEM images (Figure 4), and indirectly by ICP-OES quantification of elemental magnesium and calcium and measurement of dry mass percentage (Figure 1).

The amount of mineral formed was highest when the mineralization medium contained only calcium, i.e., in sample group WPI_U_MA. Furthermore, when both calcium and magnesium were present in the mineralization medium, calcium was preferentially incorporated into mineral formed. These results were consistent with previous studies on urease-mediated mineralization of GG hydrogels [14]. This effect was also observed for CaP formation and can be explained by the mechanism proposed by Martin and Brown [29]. Accordingly, magnesium ions in solution are more hydrated than calcium ions, thus undergoing dehydration more slowly, which results in their adsorption on CaCO_3_. Subsequently, they are not included in the carbonate mineral and remain on the surface. As a result, the total amount of magnesium the mineral formed is lower.

Marine research on exoskeletons of marine invertebrates mineralized with CaCO_3_ has revealed similar effects. In studies in which the effect of the Mg:Ca elemental ratio in seawater on the incorporation of magnesium into marine invertebrate exoskeletons and non-skeletal precipitation was investigated [30,31], preferential incorporation of calcium was reported.

A decrease in crystallinity in the order WPIU_MA > WPIU_MB > WPIU_MC was observed using Raman spectroscopy (Figure 2B), XRD (Figure 3) and SEM (Figure 4). This can also be explained by the mechanism proposed by Martin and Brown (see above), whereby magnesium ions dehydrate more slowly, and hence, are not included in the carbonate mineral and remain on the surface [29]. XRD results suggested that small amounts of calcium magnesium carbonate may have formed in WPIU_MC samples (Figure 3). Raz et al. (2000) proposed that calcium magnesium carbonate forms via an amorphous precursor phase, into which hydrated magnesium ions can be more easily incorporated in the amorphous precursor phase, and hence, are more likely to be incorporated into the final, more crystalline calcium magnesium carbonate phase [32]. Furthermore, magnesium may stabilize the amorphous precursor phase; magnesium has been reported to ‘poison’ calcite formation by binding to the surface of calcite nuclei and inhibiting further crystal growth as a result of its hydration [33].

SEM images (Figure 4) revealed differences in the morphologies of mineral deposits formed in WPI hydrogels in this study and those formed in GG hydrogels in previous work [14]. Hydrogels are 3D crosslinked polymer networks with entrapped water. The mechanism of WPI hydrogel formation at elevated temperature and pressure involves denaturation of its main component β-lg, leading to unfolding of the molecule and the formation of disulphide bonds between β-lg molecules and the formation of a 3D crosslinked network [34].

Differences between the structures of WPI hydrogels and GG hydrogels may influence the type of mineral formed. The concentration of polymer in WPI hydrogels in this study (50% (*w*/*v*)) is much higher than in the GG hydrogels used in previous works (0.7% (*w*/*v*)) [10,14,15]. A higher concentration of polymer would lead to smaller pores in the polymer network. In turn, this would hinder diffusion of mineralization medium through the hydrogel, which would lower the amount of mineral formed. Another consequence of small pores is hindrance of the formation of large crystals. Different degrees of compactness of the mineralized hydrogels were observed (Figure 4D–F), which may be due to the differences in the sizes of the mineral deposits. A higher concentration of polymer might however promote mineral formation; diffusion of enzyme out of the hydrogel would be impeded, and also, more polymer chains mean more potential binding sites for calcium or carbonate ions, which may serve as nucleation sites for crystal growth.

There is no obvious correlation between amount of Ca and Mg released (Figure 6) and cell proliferation (Figure 5). This demonstrates that the amounts of Ca and Mg released are non-cytotoxic and may be considered harmless. Ca concentrations of 10 mM and above have been reported to be toxic for osteoblasts, while concentrations in the range 2–4 mM have been reported to be beneficial for proliferation [35]. The Ca (and Mg) concentrations in cell culture media are in the range 0–10 mM (Figure 6), however, no obvious positive effect was observed. It is to be expected that protein is released from WPI hydrogels; one may speculate that the released protein may be binding Ca and Mg and hindering any stimulatory effect of these ions.

Previous work on GG hydrogels mineralized with calcium and magnesium carbonates by enzymatic mineralization demonstrated that hydrogels mineralized with calcite improved the adhesion and proliferation of osteoblast-like MC3T3-E1 cells, and that hydrogels mineralized with calcite containing small amounts of magnesium had no appreciable negative effect [14]. Similarly, a previous study on GG hydrogels mineralized with calcium and magnesium carbonates by alternate soaking in solutions of calcium/magnesium and carbonate ions also demonstrated comparable proliferation and differentiation of osteoblast-like MC3T3-E1 cells on hydrogels mineralized with predominantly calcite and vaterite and those mineralized with predominantly calcite and vaterite containing small amounts of magnesium [15].

The results of this study suggest that magnesium as a dopant of CaCO_3_ does not provide an appreciable positive biological effect on cell viability, in contrast to reports of a positive effect of magnesium as a dopant of CaP. The reasons for this remain unclear and are outside the scope of this work. In this study, no appreciable effect of mineralization on swelling was observed. However, such effects are worthy of investigation in future work.

## 4. Materials and Methods

### 4.1. Production of Urease WPI Hydrogels Containing Urease

WPI hydrogels were produced as previously described [2,3,4]. Briefly, WPI powder (Davisco, Le Sueur, MN, USA) was added to ddH_2_O for a final concentration of 50% (*w*/*v*). The solution was incubated in an ultrasonic bath for 30 min to ensure the powder had fully dissolved. Urease extracted from Canavalia ensiformis (Sigma Aldrich, U1500, Poole, UK) was added to WPI solution at a concentration of 10 mg urease/mL solution prior to the solution being heated to 70 °C for 8 min for gelation.

### 4.2. Mineralization of Urease WPI Hydrogels

The urease WPI hydrogels were mineralized using three different media at room temperature over 7 days. In these media, the concentration of urea (0.17 M) was kept constant whilst the ratio of CaCl_2_:MgCl_2_ was varied (Table 1). Unmineralized control samples were prepared in a similar fashion by incubating urease-free WPI hydrogels in the mineralization media.

### 4.3. Determinination of Mineral Formation and Elemental Composition

To assess the extent of mineral formation, the samples were dried at 60 °C for 48 h. Samples were weighed before and after the drying process to calculate the dry mass percentage, or (weight after drying/weight before drying) × 100%, which the mass percentage is attributable to polymer and mineral and not water and serves as a measure of extent of mineralization.

Inductively coupled plasma optical emission spectroscopy (ICP-OES) was used to determine the calcium and magnesium content of samples using a 5100 Synchronous Vertical Dual View spectrometer (Agilent, Cheadle, UK). Prior to analysis, solid samples were dried and ground before being diluted in 1% nitric acid and diluted 10-fold. No additional steps were necessary for liquid samples (cell culture medium), which were diluted in 1% nitric acid immediately.

### 4.4. Physiochemical and Morphological Characterisation: Raman, XRD, SEM, FTIR

Samples were dried at 60 °C for 48 h prior to analysis. Raman spectra were collected using a confocal Raman system (InVia, Renishaw plc, Wotton-under-Edge, UK) equipped with a near infrared laser. A laser power of ~15 mW was used to prevent any damage to the samples. Spectral collection exposure time was set to 1 s with one acquisition. Spectra were collected from each sample over the spectral range of 60–1800 cm^−1^. Pre-processing, such as baseline correction, was carried out using Wire 4.0 software (Renishaw plc, Wotton-under-Edge, UK).

X-ray diffraction (XRD) measurements were performed with a Rigaku SmartLab 9 kW with a DteX-250 detector and a Cu rotating anode source (Rigaku, Tokyo, Japan). Diffractometer was set to 45 kV and 200 mA. Five degree soller slits and 2 theta scans in Bragg–Brentano configuration were used.

Morphological characterization of hydrogels was performed using scanning electron microscopy (SEM). Firstly, samples were coated with a 9 nm layer of gold for 3 min at 20 mA and 1 × 10^−1^ mBar using a Quorum Q150RES sputter coater (Quorum Technologies Ltd, Lewes, UK). SEM analysis was performed using a secondary electron detector JSM-7800F (Jeol UK Ltd., Welwyn Garden City, UK). Images were acquired with an accelerating voltage of 5 kV at a working distance of about 10 mm.

The chemical structure of samples was examined using Fourier transform infrared spectroscopy (FTIR) (Agilent Technology, Cheadle, UK) in Attenuated Total Reflectance (ATR) mode. Spectra were collected in the 500–4000 cm^−1^ spectral range with a resolution of 4 cm^−1^ and an average of 8 scans.

### 4.5. Cell Culture and Cell Viability and Release of Calcium and Magnesium from Hydrogels into Cell Culture Medium

The mouse osteoblast MC3T3-E1 cell line (ATCC^®^ CRL-2594™, Gaithersburg, MD, USA) was routinely cultured in MEM-α medium supplemented with 10% FBS and incubated in a humidified 5% CO_2_ environment at 37 °C. Once 80% confluent, cells were trypsinized and used for cytotoxicity testing. Cell tests were carried out in accordance with the International Organization for Standardization (ISO) norm ISO 10993-5.

In 96 well polystyrene plates, 4 × 10^3^ MC3T3-E1 cells were seeded on WPI hydrogels and incubated for 24 h to allow the cells to adhere. MC3T3-E1 seeded WPI hydrogels were washed with PBS (pH 7.4) before AlamarBlue (10% in media) was added and allowed to incubate for 2 h. Fluorescence analysis was performed using an excitation wavelength of 530 nm and an emission wavelength of 590 nm. Controls lacking cells were used to determine background fluorescence which was subsequently subtracted from cell viability results. Cell viability was measured after 24 h and 72 h.

To study release of calcium and magnesium, WPI hydrogels were incubated for 24 h or 72 h in cell culture medium under sterile conditions in the absence of cells. Calcium and magnesium concentrations in media were determined using ICP-OES, as described above

### 4.6. Statistical Analyses

Student’s *t*-test was applied to determine statistical significance using Excel software. The results of average weight, dry mass percentage, calcium and magnesium concentration and biological tests were analyzed. A two-tailed unpaired *t*-test with 95%, 99% and 99.9% confidence intervals was considered statistically significant if *p* < 0.05 (*), *p* < 0.01 (**) and *p* < 0.001 (***).

## 5. Conclusions

WPI hydrogels were enzymatically mineralized with an inorganic phase consisting of CaCO_3_ or magnesium-enriched CaCO_3_. Calcium was incorporated into the mineral formed to a greater extent than magnesium. Increasing the concentration of magnesium in the mineralization medium led to a reduction in the mineral formed. These observations were confirmed by dry mass percentage and ICP-OES. Moreover, increasing the amount of magnesium in medium resulted in a less crystalline structure of the mineral formed in hydrogels as shown by XRD and Raman spectroscopy results. The type of the carbonate phase detected in all hydrogels with urease was mainly calcite, which was confirmed by SEM morphology observation, XRD and FTIR analysis. There were also some vaterite deposits detected in all hydrogels mineralized by urease. XRD analysis showed that in hydrogels incubated in mineralization medium MC with equimolar concentrations of calcium and magnesium, some calcium magnesium carbonate was also present. Biological studies revealed that hydrogels were not cytotoxic. Hydrogels mineralized by urease had similar cell viability, which amounted to 74%. The presence of magnesium in the mineral formed did not promote or inhibit cell metabolic activity.

Further work should focus on biological studies concerning cell differentiation and in vivo implantation in order to determine the influence of CaCO_3_ and magnesium-enriched CaCO_3_ on bone tissue regeneration.

## Figures and Tables

**Table 1:** Mineralization media composition

Medium	Concentration (mol/dm^3^)			Ratio
	CaCl_2_	MgCl_2_	Urea	
MA	0.2700	0	0.1700	1:0
MB	0.2025	0.0675	0.1700	0.75:0.25
MC	0.1350	0.1350	0.1700	0.5:0.5
